# Random lasing in human tissues embedded with organic dyes for cancer diagnosis

**DOI:** 10.1038/s41598-017-08625-3

**Published:** 2017-08-21

**Authors:** Yu Wang, Zhuojun Duan, Zhu Qiu, Peng Zhang, Jianwei Wu, Dingke Zhang, Tingxiu Xiang

**Affiliations:** 1 School of Physics and Electronic Engineering, Chongqing Normal University, Chongqing, 401331 China; 2 Chongqing Key Laboratory of Molecular Oncology and Epigenetics, The First Affiliated Hospital of Chongqing Medical University, Chongqing, China

## Abstract

Various nanostructures found in biological organisms are often complex and they exhibit unique optical functions. This study surprisingly found that typical random lasing occurs in cancerous human tissues embedded with the nanotextured organic dye 4-(dicyanomethylene)-2-tert-butyl-6-(1,1,7,7- tetramethyljulolidyl-9-enyl)-4H-pyran (DCJTB). Hematoxylin and eosin stain images show that there are more laser resonators in cancerous tissues, caused by a large number of disordered scatters. It is also noteworthy that the random lasing thresholds were found to relate to the tumor malignancy grade. Consequently, the resulting typical random lasing resonators differ between cancerous tissues in different malignancy grades. Further studies are warranted to investigate tissue optical spectroscopy in the field of cancer diagnostics.

## Introduction

In recent years, random lasers (RLs) have attracted considerable attention because of their unique laser oscillation phenomena without a clear cavity structure^1–6^. Unlike conventional lasers, which occur within carefully configured resonant cavities, a RL is the simplest source of stimulated emission without cavity, whose feedback mechanism is based on randomly placed nanoparticles embedded in the active material providing distributed optical feedback for lasing action^7, 8^. Random lasing has been divided into two main operational regimes: the so-called “resonant” and “non-resonant” emissions^9, 10^. For resonant emission, based on back-scattering albedo caused by recurrent scattering processes, if a light wave propagates in a closed-loop path and forms a loop resonator, the light wave interference will introduce coherence and feedback, leading to lasing action^11^. There is appearance of a multi peak with randomly placed narrow frequency modes in the spectrum^7, 9^. However, for non-resonant emission, the spectrum tends to be a single peaked spectrum with a few nanometer width, which has been understood as strong coupling between adjacent modes in simulation. The “non-resonant” regime is normally consisted of a large number of modes mutually coupled together, resulting in a broad single-peaked spectrum^12, 13^.

In general, lasing action in RLs is usually obtained with a spatially distributed feedback, where scattering particles and active material share the same region of space, obtaining an amplifying scattering medium. Recently, López *et al*.^14^ proposed a different approach, in which gain region and feedback elements are spatially separated. Two random agglomerations of highly scattering particles are placed near the edges of an optically pumped active medium, obtaining coherent random lasing emission. Therefore, a spatially localized feedback mechanism has been proposed.

For random lasers, two components are necessary: disordered scatters and gain materials. Random lasing action has been demonstrated in various disordered optical gain media, such as organic molecules^15^, semiconductor powders^16, 17^, and metallic nanostructures^18^. Various applications have been proposed, such as material labeling, planar display^19^, and sensors^20^.

In recent years, biological sources as scatters have been utilized in the field of random lasers^21^ and a potential application of tumor detection has been proposed^22^. The detection principle is based on the interaction of light waves with biological tissues, where the light wave interference effects of recurrent multiple scattered light will introduce coherence and feedback, leading to lasing action. Since the disorder begun to play an important role for randomly distributed feedback, then the consequent laser emission spectrum was strongly dependent on the illuminated spot of the sample with a specific scatters configuration^11, 23^. On the other hand, it has been recently demonstrated that the cell morphology of cancerous tissue is more disordered compared to healthy tissue, which indicates that there are more laser resonators in the cancerous tissue due to more scatters and/or excess disorder. Polson *et al*. reported that biological tissues, including human tissues, can support coherent random lasing when infiltrated with a concentrated laser dye solution^24^. They found that malignant tissues show many more laser lines compared to healthy tissues taken from the same organ. However, studies investigating the effects of random laser emission on cancerous tissues with different malignancy grades have not been conducted.

In this study, we found that biological tissues marked with laser dyes can serve as a convenient platform for the exploration of random lasing action. Using tissue optical spectroscopy based on random lasing detection, the optical pumped random lasing properties were investigated in healthy and cancerous human tissues with different malignancy grades. By comparing the hematoxylin and eosin (HE) stain images, random lasing emission spectra and lasing thresholds, we could distinguish between healthy and cancerous human tissues, and malignant tissues in different grades.

## Methods

### Materials and reagents

Primary breast tumor samples and paired surgical margin tissues were collected from the First Affiliated Hospital of Chongqing Medical University, China. All tumor samples were reviewed by pathologists to ensure the percentage of tumor cells was over 70%. Clinical information was collected. The study was approved by the ethics committee of the hospital (approval notice: 2010/2012(23)). All the experiments which uses the tissue samples and further publication of identifying information/images have been informed the involving human participants and obtained their consent. All experiments were performed in accordance with relevant guidelines and regulations.

There were more than 120 tissue sections for lasing detection, which were divided into two groups: breast tumor tissues and healthy tissues which are paired surgical margin tissues. The tumor tissue sections were further subdivided into three subgroups: malignancy grade I, malignancy grade II, and malignancy grade III, according to the pathological analysis. Before being embedded with the laser dye, all tissue sections were treated through a deparaffinning and hydrating process. During this process, the selected tissue sections were initially kept in the oven at 60 °C for 4 hours, then they were soaked in xylene solvent for 20 minutes, and subsequently transferred into fresh xylene and soaked for a further 20 minutes. During the second part of the deparaffinning and hydrating process, the tissue sections were hydrated with 100% ethanol for 5 minutes, followed by 95% ethanol for 5 minutes, 80% ethanol for 5 minutes and 60% ethanol for 5 minutes. The third stage involved rinsing the tissue sections three times with distilled water, five minute each time. During the last part of the process, the de-paraffinized tissue sections were dried in a vacuum chamber at room temperature.

### Devices

**Figure 1:**
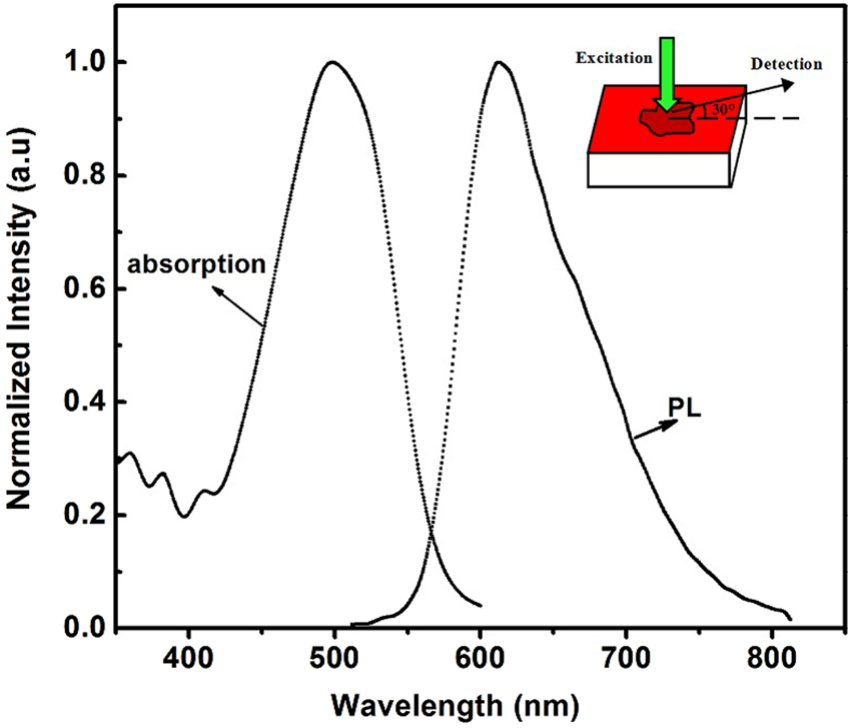
Absorption and PL spectra of DCJTB molecules. The inset shows the schematic diagram of the sample.

The tissue samples were soaked in the laser dye4-(dicyanomethylene)-2-tert-butyl-6-(1,1,7,7–tetramethyljulolidyl-9-enyl)-4H-pyran (DCJTB), which, as the gain media, has a strong absorption band in the green spectral range and a fluorescence band in the red spectral range. Figure 1 shows the normalized absorption and photoluminescence (PL) spectra of DCJTB. The absorption spectrum at 498 nm and PL spectrum at 600 nm originate from the absorption and emission of DCJTB molecules. For comparison, a bare DCJTB doped polystyrene (PS) film without biological tissue was also fabricated by spin-coating onto an untextured quartz substrate. All the samples were dried in a vacuum.

## Methods

The experimental setup to investigate the laser action followed ref. 25. The pumping source was a frequency tripled Nd doped yttrium aluminum garnet laser (Spectra-Physics) delivering 10 ns pulses at 532 nm with a 10 Hz repetition rate. The output pulse energy of the pumping laser was controlled using neutral density filters. An adjustable slit and a cylindrical lens were used on the beam splitter in order to shape the beam into a narrow stripe with a continuously varied length on the sample film. As the schematic diagram shown in the inset of Fig. 1, the excitation beam was incident in the vertical direction with respect to the surface and the laser strip at the tissue surface were 0.8 mm wide and 5 mm long. The output signals were detected at an angle of 30 degrees with the horizontal direction by a fiber-coupled, charge-coupled device spectrometer (Jobin Yvon-SPEX CCD3000). The pumped energies from the laser were measured by using a calibrated laser power and energy meter (Gentec). The absorption spectra were measured with a Lambda 345 UV-visible spectrometer (Perkin-Elmer), and the PL spectra were measured with an LS-50B luminescence spectrometer (Perkin-Elmer). The morphology and structure of the biological tissues were characterized by hematoxylin and eosin (HE) stain.

### Data availability statement

The authors declare that data in our manuscript are available.

## Results and Discussion

### Morphology Identification

**Figure 2:**
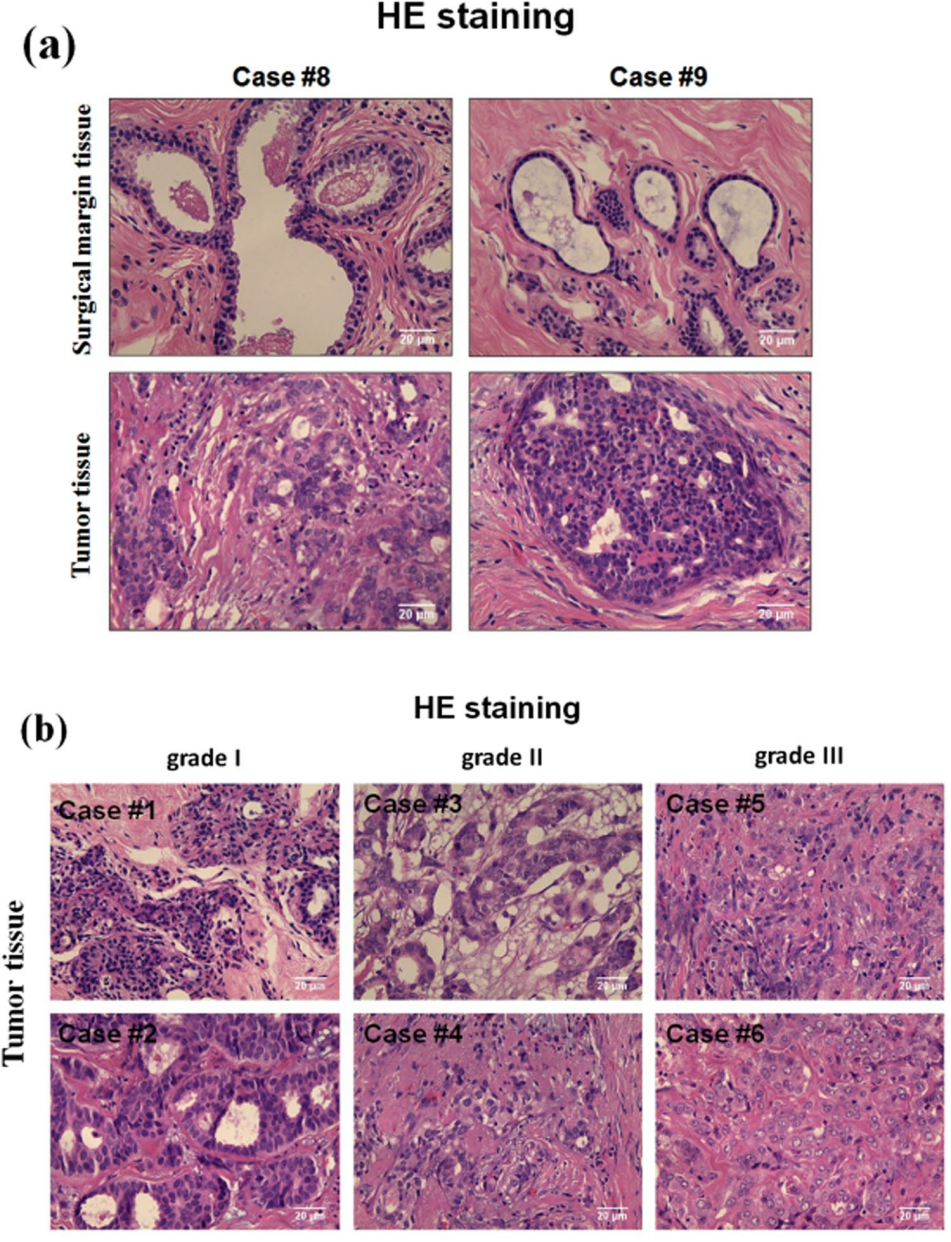
(**a**) The HE stained microscopic image of surgical margin tissues and tumor tissues; (**b**) The HE stained microscopic image of human breast tumor tissues: malignancy grade I (case#1 and #2), malignancy grade II (case#3 and #4), and malignancy grade III (case#5 and #6).

The HE is the most widely used stain in pathology. Figure 2(a) shows the HE staining image of the primary breast tumor tissues and paired surgical margin tissues. It can be clearly seen that the spatial arrangement of the breast tumor cells is more disordered and the organizational structure is much more irregular than the paired surgical margin tissue. The HE stain features of different cancerous tissues in different malignancy grades are also shown in Fig. 2(b). The degree of the malignant transformation is closely related to the degree of disorder of the tissue structure, which leads to more laser resonators in the cancerous tissue due to more scatters.

### Random Lasing Performance

**Figure 3:**
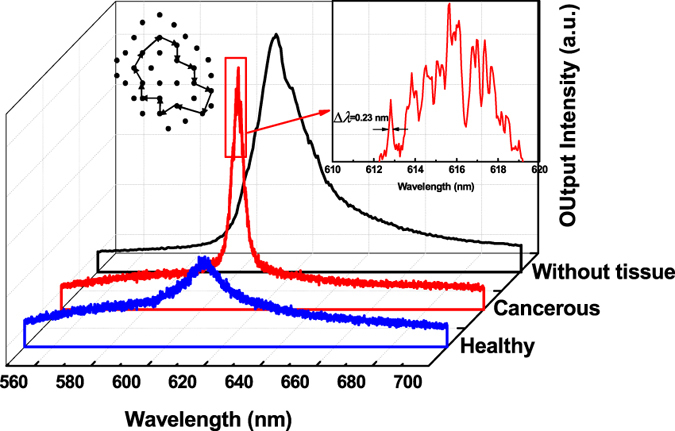
The emission spectra from the bare DCJTB doped PS film, and the emission spectra from the healthy and cancerous breast tissues marked with nanotextured DCJTB. The inset shows a portion of the spectrum of the nanotextured DCJTB embedded cancerous human tissue under higher magnification (right) and a schematic diagram showing the formation of a closed-loop path for light through multiple optical scatters (left).

Figure 3 shows the emission spectrum from the bare DCJTB doped PS film on the quartz substrate without biological tissue, and the emission spectra from the healthy and tumor tissues, marked with nanotextured DCJTB, when pumped by a pulsed source with a 0.7 mm wide and 5.7 mm long narrow strip at a pumping energy of 0.3 mW. It can be seen that the emission spectra from the bare DCJTB doped PS film and the healthy and tumor tissues differ markedly. In the case of the bare DCJTB doped PS film, only one emission peak in the red region is observed, which originates from the amplified spontaneous emission (ASE) of DCJTB molecules^26^. The full width at half-maximum (FWHM) of the emission spectrum is generally 16 nm or more. The healthy human tissues embedded with DCJTB yielded a weak single peak at this pump energy. However, in the case of the cancerous human tissue embedded with DCJTB, an additional narrowed spectrum with discrete peaks emerged. Figure 3 shows the portion of the multimode spectrum under higher magnification of the cancerous human tissue embedded with DCJTB. The spectra with more resolution corresponding for Fig. 3 are shown in Figure S1 in the Supporting information. The mode linewidth is less than 0.23 nm, which is 69 times smaller than that of the ASE peak of the DCJTB doped PS film. This indicates that the typical random lasing action has occurred in the cancerous human tissue embedded with nanotextured DCJTB^11^.

Since the concentration and thickness of the gain media is the same for all three types of samples, the difference in emissions is attributable to the different characteristics of the tissues. The degree of disordered tissue structure is the key to understanding the resulting random lasing emission. Since a disordered medium leads to random scattering, resulting in random resonators, photons will undergo multiple scattering events with some non-zero probability of being returned to their starting point, forming a closed loop^7^, as shown in the inset of Fig. 3. As clarified in the introduction, the feedback mechanism in our experiments belongs to the case of RLs with distributed feedback and strong mode coupling. This closed loop is similar to the ring cavity which is part of the entire distributed feedback structures. The feedback for lasing action is given by the ensemble of randomly distributed scattering centers embedded with the active material. There are many such closed-loop paths as the resonant cavities in the nanotextured DCJTB embedded cancerous human tissue. Along different loops, the probability of a photon scattered back to its starting point is different. For the case of cancerous human tissue scattered DCJTB:PS film, the transport mean free path can be obtained by the following equation^27^:

1 $$L=\frac{{\lambda }^{2}}{2n{\rm{\Delta }}\lambda }$$

where *L* is the transport mean free path and *n* is the effective polymer refraction index, which in our case is close to 1.65. Using the measured peak separation of Δ*λ* = 0.54 nm, we estimated *L* to be 212 *μm* for the case of cancerous human tissue scattered DCJTB:PS film. Obviously, when compared to healthy tissues, the disordered tumor tissues form more of these loops for random lasing emission.

**Figure 4:**
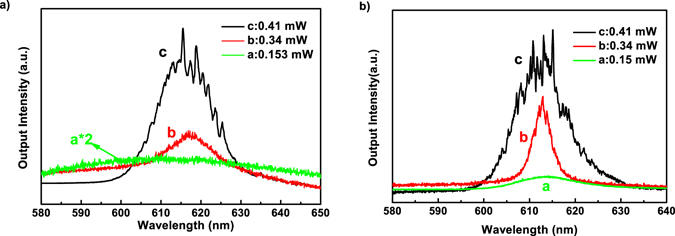
Emission spectra from the nanotextured DCJTB embedded in (**a**) healthy and (**b**) cancerous tissues of human breast at different pumped energies.

The emission spectra from the healthy and cancerous breast tissues marked with nanotextured DCJTB at different pumped energies are shown in Fig. 4 (A portion of the mission spectra under higher magnification are shown in Figures S2 and S3 in the Supporting information). It can be seen that broad emission spectra are obtained at lower pump energies. With the pump energies increasing, both healthy and cancerous breast tissues present random lasing performance because of the emergence of the discrete sharp spikes in the narrowed emission spectra. However, at the same pump energies (0.34 mW and 0.41 mW), cancerous tissues marked with DCJTB exhibit a multimode emission with the appearance of more sharp peaks. Furthermore, more laser lines in the emission spectra of the cancerous tissues are visible, compared to the healthy tissues, which has been checked by nearly 500 different emission spectra at different pump energy from the average of 120 samples. Obviously, whether the sample is cancerous or not plays a key role in the random lasing performance. This indicates that there are more laser resonators in the cancerous tissue due to more disordered scatters.

**Figure 5:**
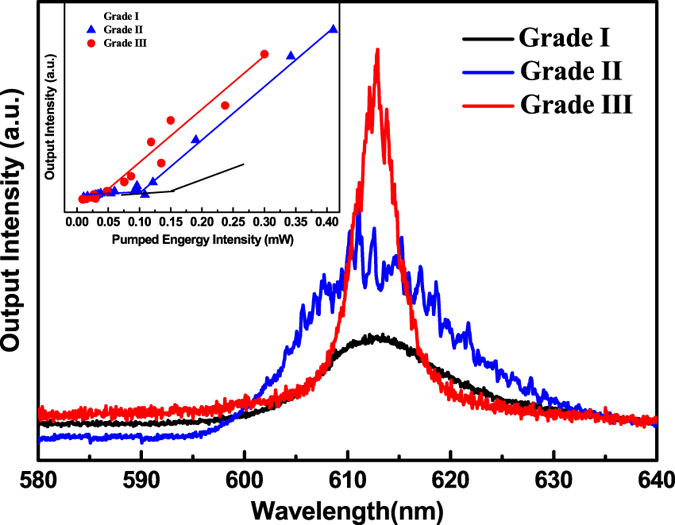
Emission spectra from grade I, II, and III cancerous tissues, marked with DCJTB, at a constant pumped intensity of 0.15 mW.

Different grades of tumors exhibit different lasing spectra with respect to the same pumped energy. Figure 5 displays the emission spectra from the nanotextured DCJTB embedded in malignant tissues in grade I, II, and III, respectively, at a constant pumped intensity of 0.15 mW (A portion of the mission spectra under higher magnification are shown in Figure S4 in the Supporting information). Obviously, with the malignant grade increasing from I to III, the output intensities and the sharp peaks gradually increase. It can be seen that the resulting spectra indeed show the dependence of the malignant grade. This dependence on the malignant grade is not difficult to understand. For a more malignant tumor tissue, the cell structure of the malignant tissue is more disordered, which indicates that there are more closed-loop paths for light in a more malignant tumor tissue due to more scatters.

The lasing threshold phenomenon is one of the most important properties to confirm lasing action. The inset of Fig. 5 compares the output emission intensity integrated over all wavelengths as a function of the pump intensity of the different malignancy grades of the tissues. The laser thresholds are clearly observed for the three grades. There is an abrupt change in the slope of the output energy versus input energy curves, followed by a linear increase of the output energy as the excitation energy. It can be seen that with increasing malignancy grade, the thresholds get lower. This is also related to the cell morphology which is more disordered when the malignancy grade increases.

**Figure 6:**
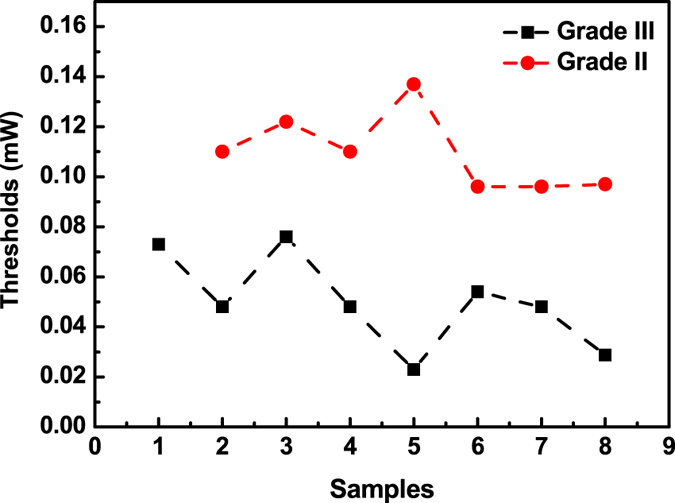
The thresholds of nanotextured DCJTB embedded in malignant tissues from 15 randomly selected samples.

To further explore the relationship of the random lasing thresholds on the malignancy grade, 15 tumor samples were randomly selected for testing. Figure 6 shows the variation of the thresholds of these cancerous tissues marked with nanotextured DCJTB. As shown in Fig. 6, no matter for the malignant tissues in grade II or grade III, there are some fluctuations in the random lasing threshold value. However, the thresholds of the malignancy grade III samples were generally lower than those of the malignancy grade II samples. Such difference between the thresholds of different grades of cancerous samples may be useful for diagnostic purposes.

## Conclusions

The study investigated random lasing action within healthy and cancerous breast tissues, infiltrated by DCJTB. The observed random lasing action arises from the human tissues’ disordered nanostructures. It is noteworthy that the random lasing spectrum relates to the tissues which are healthy or cancerous. It was found that there are more laser resonators in the cancerous tissue due to more disordered scatters. The variance in the random lasing thresholds of tissues with different malignant grades may be useful for diagnostic purposes. Further studies to investigate tissue optical spectroscopy for cancer diagnostics based on random lasing detection are warranted.

## Electronic supplementary material

Supplementary Information
