# Supplementary material for: Random lasing in human tissues embedded with organic dyes for cancer diagnosis
Source: Sci Rep. 2017 Aug 21;7:8385. doi: 10.1038/s41598-017-08625-3 (PMC5567014; doi:10.1038/s41598-017-08625-3)
Supplement: Supplementary file 1 — Supplementary Information [file 41598_2017_8625_MOESM1_ESM.doc]

**Supporting Information for**

**Random lasing in human tissues embedded with organic dyes for cancer diagnosis**

**Yu Wang^1^, Zhuojun Duan^1^, Zhu Qiu^2^, Peng Zhang^1^, Jianwei Wu^1^, Dingke Zhang^1^ & Tingxiu Xiang^2^**

***^1^School of Physics and Electronic Engineering, Chongqing Normal University, Chongqing 401331, China***

***^2^Chongqing Key Laboratory of Molecular Oncology and Epigenetics, The First Affiliated Hospital of Chongqing Medical University, Chongqing, China***


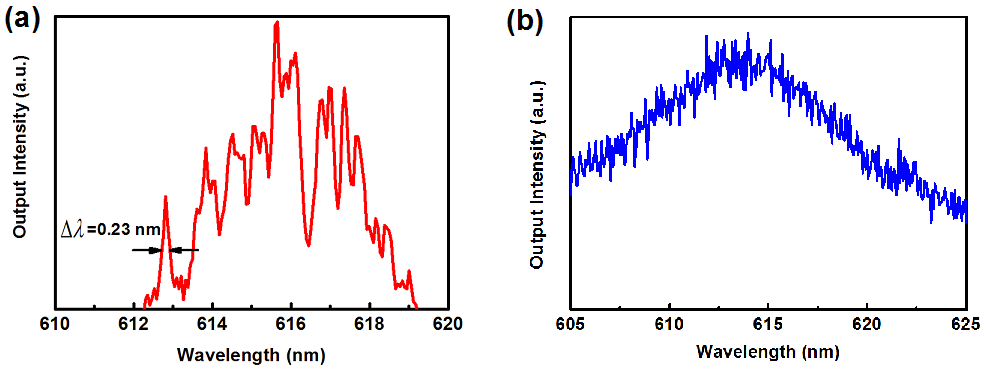


**Fig. S1**. A portion of the spectrum of the nanotextured DCJTB embedded cancerous human tissue (a) and healthy tissue (b) under higher magnification, which are corresponding for Fig.3 in the revised paper.


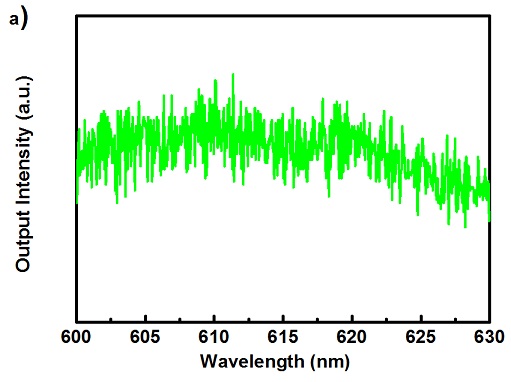


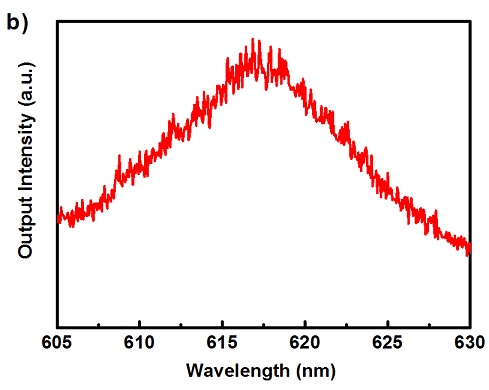


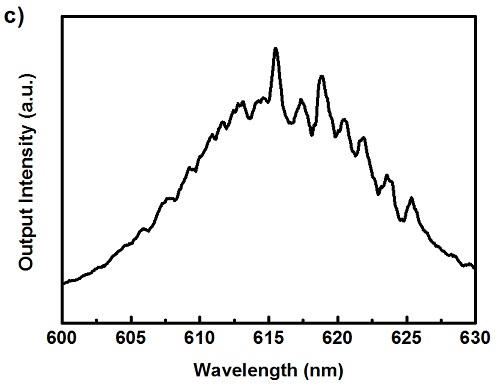


**Fig. S2**. A portion of the mission spectra under higher magnification from the nanotextured DCJTB embedded in healthy tissues of human breast at different pumped energies a) 0.153 mW, b) 0.34 mW, c) 0.41 mW, which are corresponding for Fig.4a) in the revised paper.


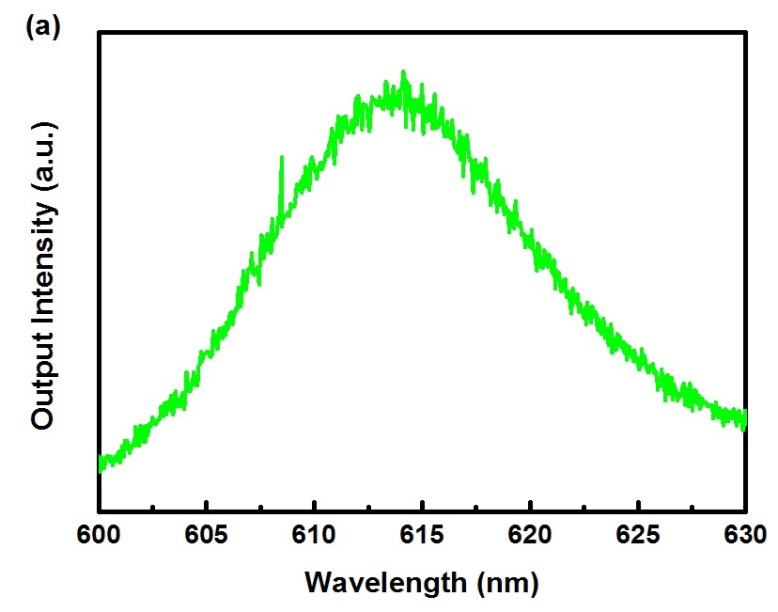


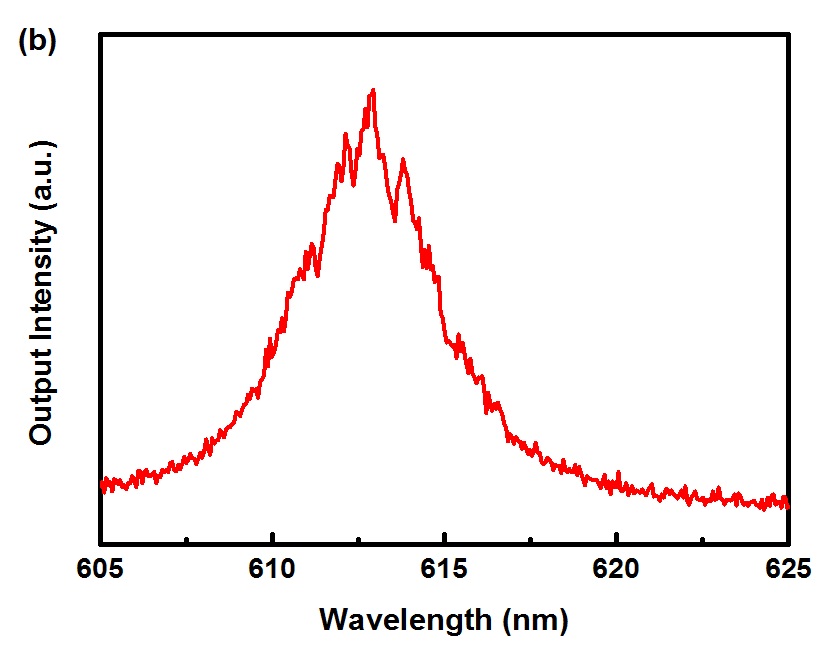


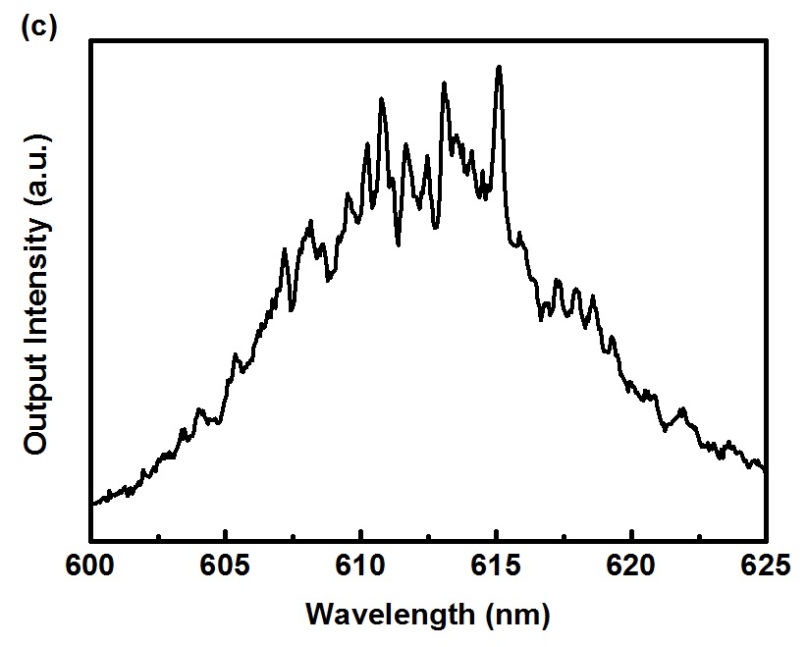


**Fig. S3**. A portion of the mission spectra under higher magnification from the nanotextured DCJTB embedded in cancerous tissues of human breast at different pumped energies a) 0.15 mW, b) 0.34 mW, c) 0.41 mW, which are corresponding for Fig.4b) in the revised paper.


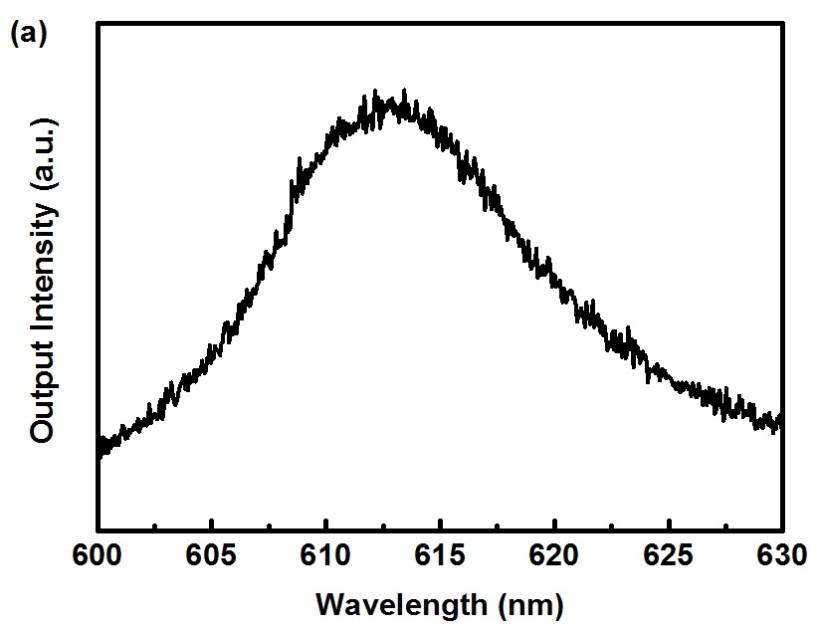


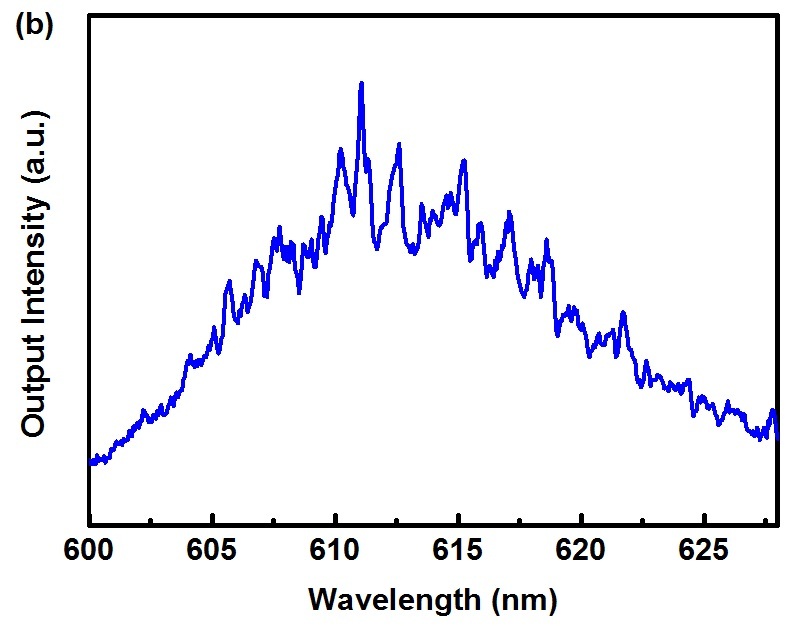


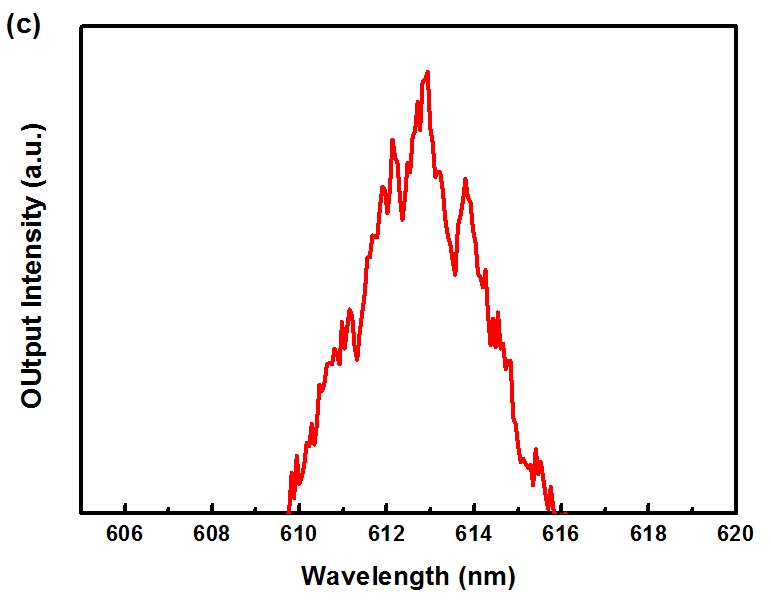


**Fig. S4**. A portion of the mission spectra under higher magnification from (a) grade I, (b) II, and (c) III cancerous tissues scattered DCJTB:PS, which are corresponding for Fig.5 in the revised paper.
